# Comparison between superficial muscularis propria and deep muscularis propria infiltration in gastric cancer patients: A retrospective cohort study

**DOI:** 10.1097/MD.0000000000004165

**Published:** 2016-07-22

**Authors:** Wei-Han Zhang, Du He, Dan-Ni Chen, Ting-Ting Li, Xin-Zu Chen, Kun Yang, Kai Liu, Bo Zhang, Zhi-Xin Chen, Zong-Guang Zhou, Jian Kun Hu

**Affiliations:** a Department of Gastrointestinal Surgery; b Laboratory of Gastric Cancer, State Key Laboratory of Biotherapy; c Department of Pathology, West China Hospital; d West China Medical School, Sichuan University, Chengdu, Sichuan Province, China.

**Keywords:** deep muscularis propria, gastric cancer, prognosis, superficial muscularis propria

## Abstract

This study aimed to investigate the clinicopathological characteristics and survival outcomes of the subclassification of pT2 gastric cancers according to the depth of tumor involvement. We retrospectively collected clinicopathological data and survival outcomes for pT2 gastric cancer patients from 2006 to 2011. Patients were classified into the superficial muscularis propria (sMP) and deep muscularis propria (dMP) groups. Eighty-nine patients had sMP gastric cancers and 90 patients had dMP gastric cancers. The rates of lymph node metastasis for the sMP and dMP groups were 55.1% and 64.4%, respectively, *P* = 0.202. The 5-year overall survival (OS) of patients in the sMP group was significantly better than patients in the dMP group (76% vs 61%, *P* = 0.018). Multivariate analysis demonstrated that the depth of tumor invasion, lymph node metastasis, and postoperative chemotherapy were prognostic risk factors for the OS. For patients with pN0 stage tumor(s), the sMP group had a significantly better 5-year OS rate than the dMP group (92% vs 62%, *P* = 0.004); for patients with pN1–N3 stages, the 5-year OS rates were comparable between the sMP and dMP groups (64% vs 61%, *P* = 0.540). The subclassification of pT2 gastric cancer into the sMP and dMP groups can demonstrate different survival outcomes according to the lymph node status. However, the pT2 stage subclassification in the next tumor, node, metastasis (TNM) staging system is pending and requires more large sample size studies to confirm its importance.

## 1 Introduction

Gastric cancer is one of the most common malignant diseases of digestive organs in East Asian countries.^[1–3]^ Radical gastrectomy combined with appropriate regional lymphadenectomy is the primary treatment strategy for this disease.^[4,5]^ Although the treatment strategy for this disease has been developed substantially in recent years, the prognosis for gastric cancer remains dismal.^[6–8]^ Currently, the TNM staging system for gastric cancer is the most frequently used classification to evaluate the tumor stages, guide the therapeutic strategies, and predict the prognosis for gastric cancer patients. In recent years, the Union for International Cancer Control/American Joint Committee on Cancer (UICC/AJCC) 7th TNM staging manual of gastric cancer and Japanese gastric cancer classification of gastric cancer, published by the Japanese Gastric Cancer Association (JGCA), reached a consensus on the gastric cancer staging system.^[9,10]^ Both staging systems defined the pT stage of tumor based on the depth of tumor invasion as pT1a (mucosa), pT1b (submucosa), pT2 (muscularis propria), pT3 (subserosa), pT4a (serosa), and pT4b (adjacent structures).^[9,10]^ On the one hand, it is clear that the tumor size, lymph node metastasis, and survival outcomes for gastric cancer patients are closely related to the depth of tumor invasion.^[7,11]^ On the other hand, the latest TNM staging system does not define details for the pT2 stage subclassification. However, the muscularis propria of the stomach consists of 2 layers, the inner circular muscle and outer longitudinal muscle. Sun et al^[12]^ subdivided the pT2 stage into the superficial muscularis propria (sMP) and deep muscularis propria (dMP); using these, they observed that sMP stage patients had better survival outcomes than dMP stage patients and dMP stage patients had survival outcomes that were similar to the subserosa stage patients. Other previous studies defined the pT2a and pT2b groups, demonstrating that pT2 gastric cancers should be divided into the pT2a or pT2b stage to evaluate the tumor stage.^[13–15]^ However, some of the studies included the subserosa stage tumor, which should be defined as the T3 stage according to the latest TNM staging system instead of as the T2 stage as in the previous TNM staging system.^[10,16]^ Therefore, we conducted this study to evaluate gastric cancer patients with tumor cells that only invaded into the muscularis propria layer (pT2 stage) and then analyzed the clinicopathological characteristics and survival outcomes between patients with tumors invading the sMP and dMP layers.

## 2 Methods

### 2.1 Ethical statement

This retrospective study and data retrieval of this study was based on the Surgical Gastric Cancer Patient Registry in West China Hospital and approved by the Ethics Committee of West China Hospital, Sichuan University. The participants did not provide written consent, but their records were anonymized in the analysis.

### 2.2 Patients

The clinicopathological characteristics and survival data of those gastric cancer patients who consecutively underwent gastrectomy from January 1, 2006 to December 31, 2011 were retrospectively collected from the prospective gastric cancer database, West China Hospital, Sichuan University, according to the study criteria. The inclusion criteria for this study were as follows: histologically confirmed gastric adenocarcinoma, pT2N0–3M0 stages by postoperative pathological examination according to the Japanese Gastric Cancer Classification 3rd English edition,^[10]^ underwent radical gastrectomy (R0) according to the Japanese Gastric Cancer treatment guidelines,^[17]^ and the availability of a complete medical record. Those patients with a history of other malignant disease or distal metastasis (M1 stage) were excluded from this study. Additionally, to minimize the confounding factors, those patients taking preoperative adjuvant chemotherapy were also excluded.

The lesions that infiltrated into the muscularis propria layer (pT2 stage) were divided into the sMP layer and dMP layer according to the depth of tumor cell invasion by pathological examination.^[12]^ This evaluation was performed according to the configuration of the muscularis propria fibers; the transversal and longitudinal muscles layers were, respectively, the sMP and dMP layers. According to the above, the pT2 stage gastric cancer patients in the study were divided into the sMP and dMP groups.

### 2.3 Treatment strategy

Patients in our hospital underwent distal, proximal, or total gastrectomy with corresponding regional lymphadenectomy according the treatment guidelines published by the JGCA.^[17]^ The preoperative examination (enhanced abdominal computed tomography and upper gastrointestinal endoscopy) and intraoperative exploration were used to confirm the clinical tumor stages. Preoperative neoadjuvant chemotherapy was recommended to the patients with advanced tumor stages in our hospital. However, because of the potential bias introduced by the neoadjuvant chemotherapy in evaluating the depth of tumor invasion and survival outcomes, the patients who underwent neoadjuvant chemotherapy were excluded from the present study. Patients who were clinically evaluated as T1 stage underwent D1/D1+ lymphadenectomy and patients who were clinically evaluated as having advanced gastric adenocarcinoma underwent D2/D2+ lymphadenectomy. Because of the potential difference between the clinical and pathological tumor stages, the lymphadenectomy strategies were analyzed in this study. Billroth-1, Billroth-2, Roux-en-Y, and esophagogastric anastomosis were all selective digestive tract reconstruction methods according to the corresponding resection patterns and were chosen according to the doctor's preference. Postoperative adjuvant chemotherapy was recommended for those patients with confirmed advanced tumor stage or with lymph node metastasis according to the pathological examination. Combinations of fluoropyrimidine and platinum regimens were used as first-line postoperative chemotherapy treatment strategies in these patients.

### 2.4 Study parameters

Clinicopathological information, including the demographic information, surgery-related information, pathologic results and survival outcomes, such as the age (years), gender (male, female), tumor size (cm), Borrmann type (Type I–IV), differentiation degree (well, moderate, poor, and undifferentiated), tumor location (upper, middle, and lower third of the stomach) resection patterns (distal gastrectomy, total gastrectomy, and proximal gastrectomy), and lymphadenectomy degree (D1/D1+ and D2/D2+). Special pathologists in the Department of Pathology, West China Hospital were responsible for the pathological examination of the gastric adenocarcinoma patients. The pathological examination was based on the principle of the Japanese Gastric Cancer Classification 3rd English edition and UICC/AJCC 7th TNM staging manual.^[9,10]^ Capillary invasion and extranodal metastasis were analyzed and confirmed according to the Japanese Gastric Cancer Classification 3rd English edition.^[10]^

### 2.5 Follow-up information

Postoperative follow-up was recommended as routine outpatient visits for gastric cancer patients in the West China Hospital. Regular outpatient follow-up was scheduled as at every 3 months during the first 2 years and subsequently every 6 months during the last 3 years. Additionally, mail questionnaires and telephone interviews were supplementary methods of follow-up to collect individual information. Follow-up was updated on January 1, 2016. The follow-up rate and the median follow-up duration (months) were to be reported in the study. The reasons of follow-up loss were predominantly absence of outpatient visit or inaccessible contact. The events of death or any pattern of recurrence were recorded during the follow-up and analyzed in the survival comparisons.

### 2.6 Statistics

Statistical analysis was performed using SPSS statistics software, version 20.0 (SPSS, Chicago, IL). The ranked variables were assessed with the Mann–Whitney *U* test. The categorical variables were evaluated with the Pearson Chi-square test. Survival outcomes were reported to the Kaplan–Merrier method, log-rank test. The 5-year overall survival (OS) rate was calculated using the life table test. Multivariate adjusted factor survival analysis was performed using Cox proportional hazard modeling. The hazard ratio (HR) and 95% confidence interval (95% CI) were used to present the univariate and multivariate survival analysis results. Two-tailed *P* values less than 0.05 were considered statistically significant.

## 3 Results

### 3.1 Demographic and pathologic information

Finally, a total of 189 pT2 gastric adenocarcinoma patients from January 1, 2006 to December 31, 2011 fulfilling the inclusion/exclusion criteria were included in the present study for analysis. In this study, 89 patients were in the sMP group and 90 patients in the dMP group according to the postoperative pathological examination. For general clinical characteristics (Table 1), there were no significant differences between the sMP and dMP groups in terms of the age (*P* = 0.774), gender (*P* = 0.830), resection pattern (*P* = 0.072), lymphadenectomy strategy (*P* = 0.503), Borrmann type (*P* = 0.309), and histological grade (*P* = 0.312). The patients in the dMP group had a larger tumor size (*P* = 0.006) and a higher number of tumors located in the middle third of the stomach (*P* = 0.008) compared to patients in the sMP group. The number of examined lymph nodes was comparable between the sMP and dMP groups (25.3 ± 11.8 vs 26.8 ± 11.6, *P* = 0.379). However, the dMP group had a higher number of positive lymph nodes than the sMP group (3.6 ± 5.7 vs 2.1 ± 2.9, *P* = 0.027). It is interesting that the proportion of patients with lymph nodes metastasis (*P* = 0.202) and postoperative N stage (*P* = 0.228) were comparable between the sMP and dMP groups (Table 2). Others pathological variables, such as extranodal metastasis (*P* = 0.711) and the capillary invasion status (*P* = 0.597), were also comparable between the 2 groups. Meanwhile, the ratio of patients taking postoperative adjuvant chemotherapy was comparable between the sMP and dMP groups, 29.2% vs 25.6%, respectively, *P* = 0.583.

**Table 1:**
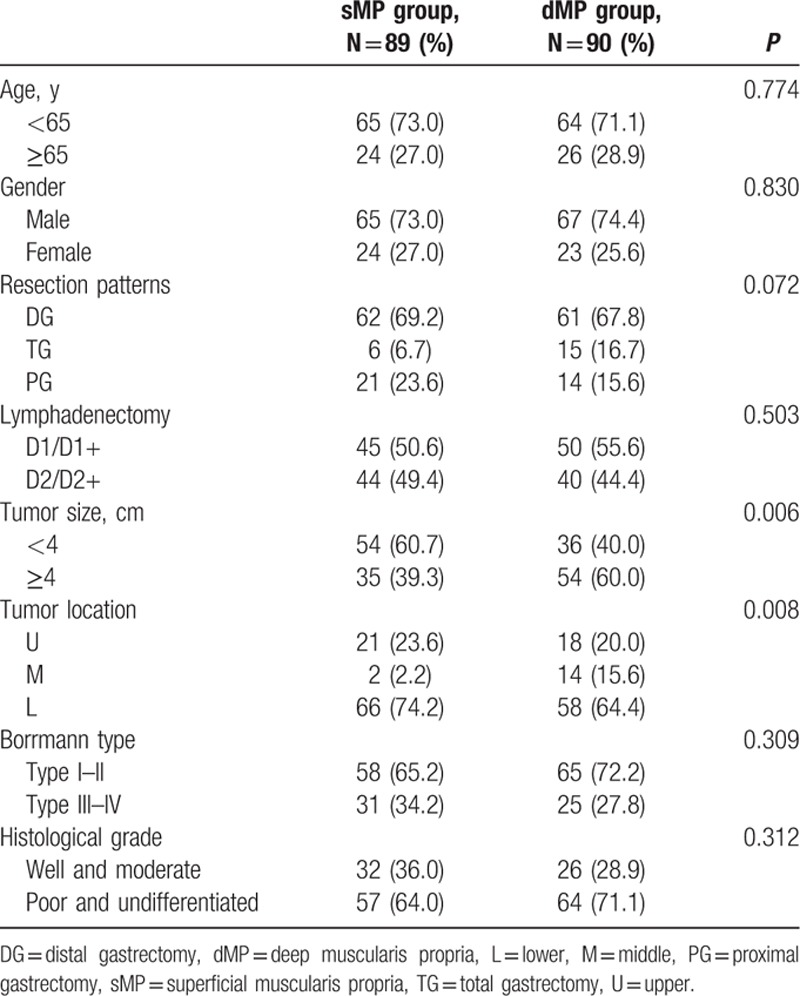
General clinical pathological characteristics of patients.

**Table 2:**
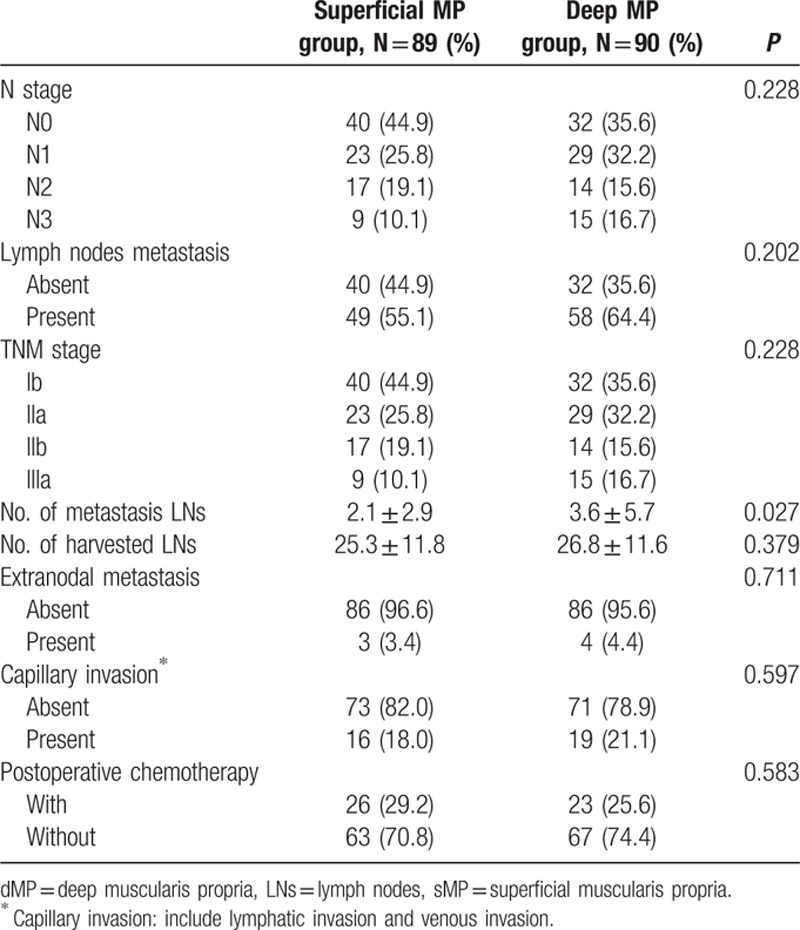
Postoperative pathologic results of patients.

### 3.2 Follow-up and survival information

**Figure 1:**
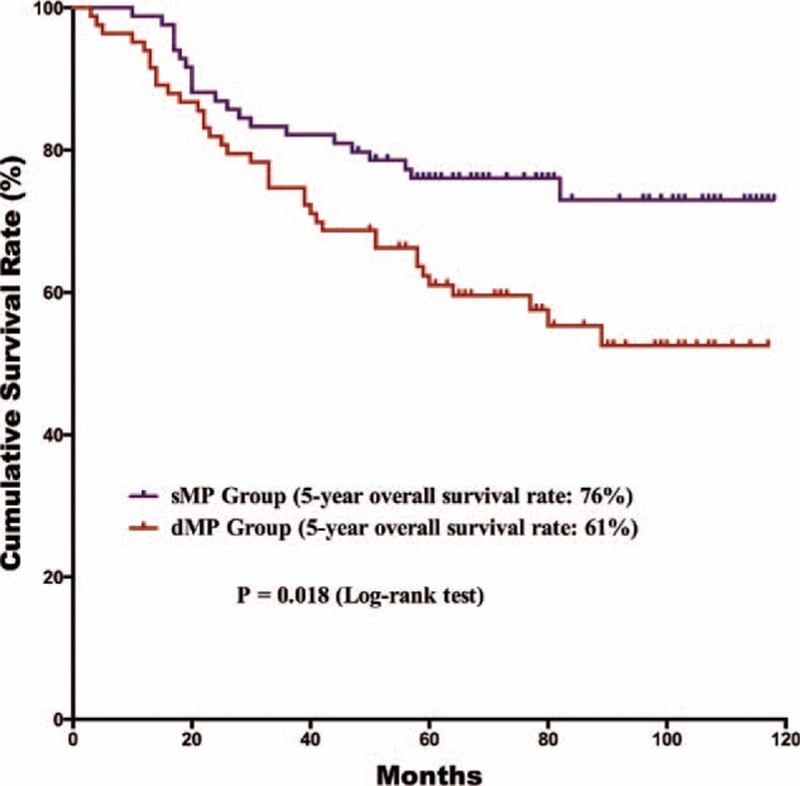
Comparison of the survival outcomes for all gastric cancer patients with sMP and dMP gastric cancers. The 5-year overall survival rate of patients with sMP tumors was better than for patients with dMP tumors (76% vs 61%, *P* = 0.018).

**Figure 2:**
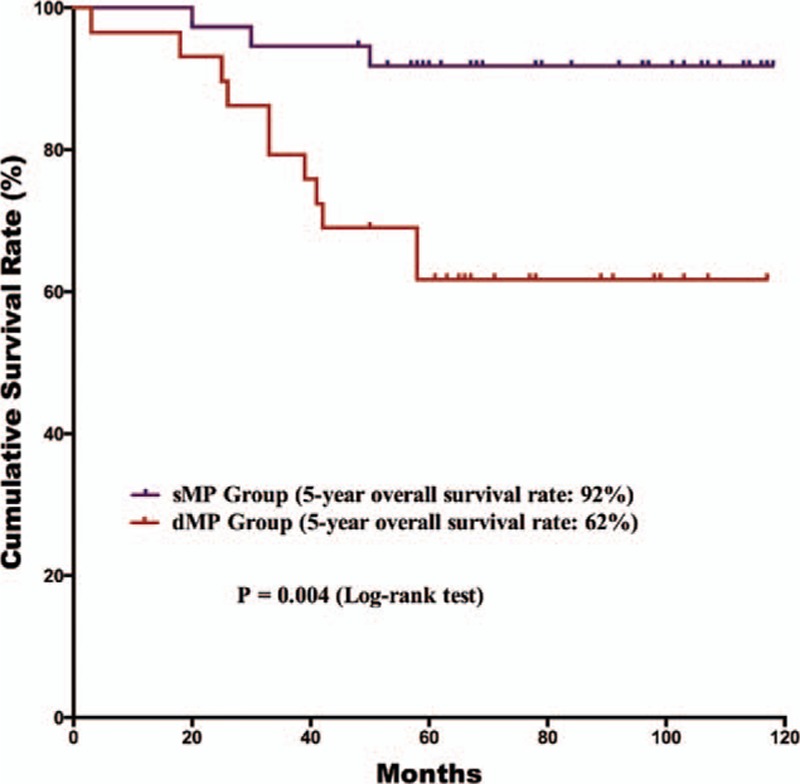
Comparison of the survival outcomes for pN0 gastric cancer patients with sMP and dMP gastric cancers. The 5-year overall survival rate of pN0 patients with sMP tumors was better than patients with dMP tumors (92% vs 62%, *P* = 0.004).

**Figure 3:**
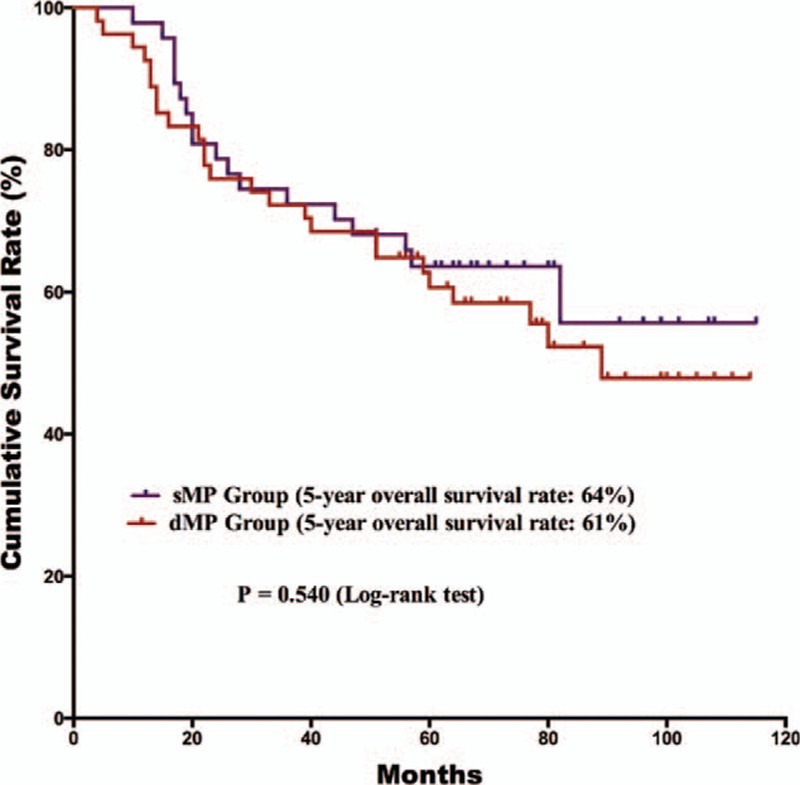
Comparison of the survival outcomes for pN1–3 gastric cancer patients with sMP and dMP gastric cancers. The 5-year overall survival rate of pN1–3 patients was comparable between the sMP and dMP groups (64% vs 61%, *P* = 0.698).

**Figure 4:**
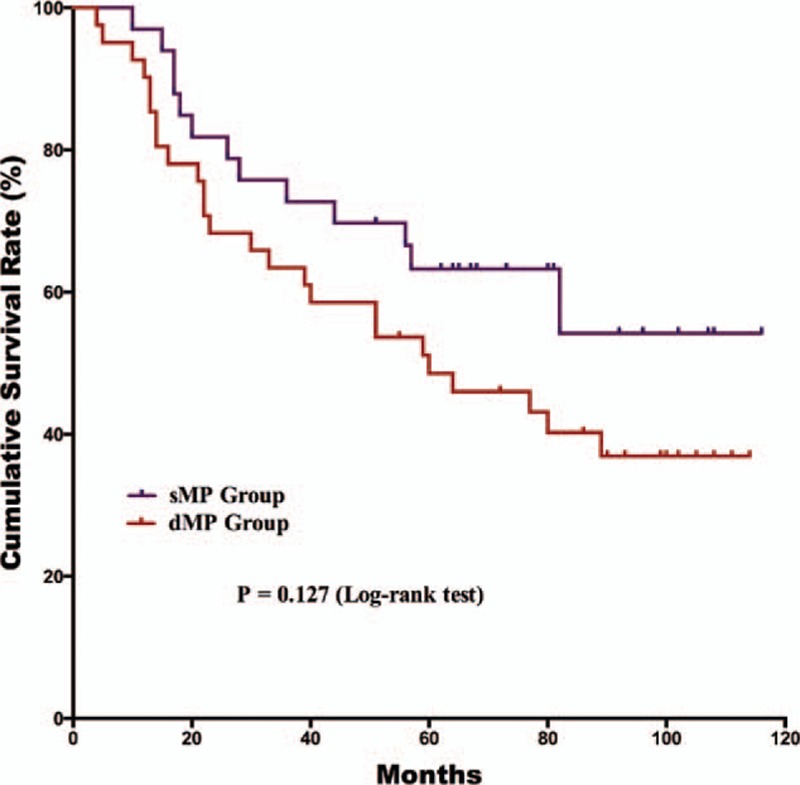
Comparison of the overall survival outcomes for pN1–3 gastric cancer patients with sMP and dMP gastric cancers who did not undergo postoperative chemotherapy (*P* = 0.127).

**Figure 5:**
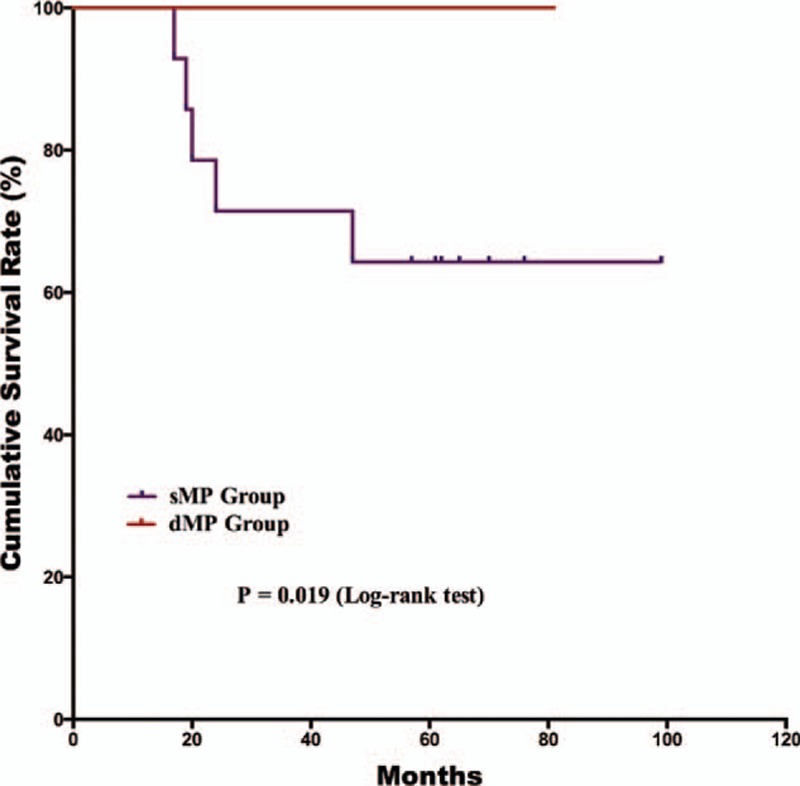
Comparison of the overall survival outcomes of pN1–3 gastric cancer patients with sMP and dMP gastric cancers who underwent postoperative chemotherapy (*P* = 0.019).

The postoperative follow-up information was updated on January 1, 2016. Finally, 12 of the 179 patients were lost to follow up in the postoperative period; the follow-up rate was 93.3% and median follow-up duration was 65 months with a range of 3 to 118 months. For the survival outcomes, patients in the sMP group had a significantly better 5-year OS rate than the dMP group (76% and 61%, *P* = 0.018, respectively) (Fig. 1). Univariate and multivariate survival analyses were conducted to explore the potential prognostic risk factors for pT2 gastric cancer patients (Table 3). According to the univariate analysis, the depth of tumor invasion (sMP vs dMP, 95% CI: 1.103–3.238), lymph node metastasis (absent vs present, 95% CI: 1.307–4.375), extranodal metastasis (absent vs present, 95% CI: 1.229–7.720), and postoperative chemotherapy (with vs without, 95% CI: 1.268–5.664) were poor prognostic risk factors for the OS. The multivariate analysis revealed that the depth of tumor invasion (sMP vs dMP, 95% CI: 1.000–2.956), lymph node metastasis (present vs absent, 95% CI: 1.184–3.987), and postoperative chemotherapy (present vs absent, 95% CI: 1.228–5.494) were independent prognostic risk factors for the OS. Therefore, we conducted subgroup survival analysis between the 2 groups depending on the lymph node metastasis status (Figs. 2 and 3). For patients without lymph node metastasis (pN0 stage), the sMP group had a significantly better 5-year OS rate than the dMP group (92% vs 62%, *P* = 0.004). For patients with lymph node metastasis (pN1–N3 stages), the 5-year OS rates of the sMP and dMP groups were comparable (64% vs 61%, *P* = 0.540). Additionally, the subgroup analyses of the postoperative chemotherapy status in the nodal metastasis patients were compared between the sMP and dMP groups (Figs. 4 and 5).

**Table 3:**
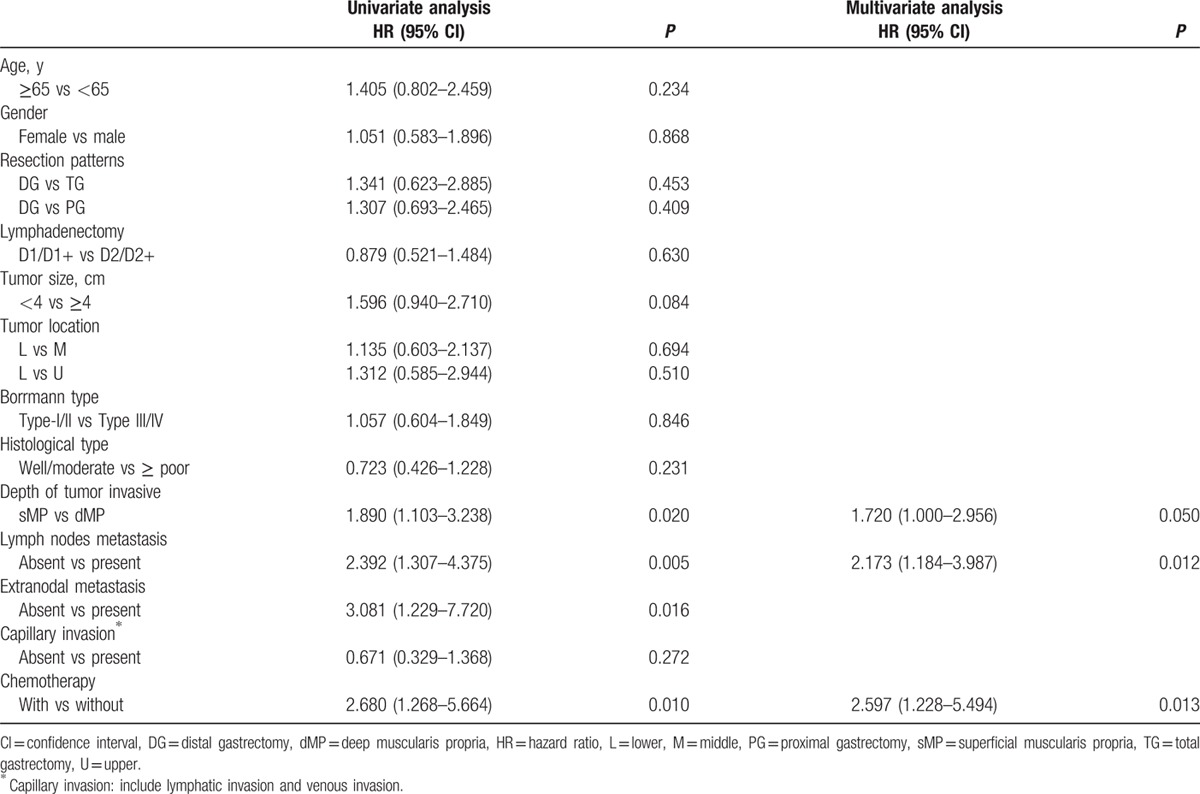
Univariate and multivariate analyses of overall survival according to clinicopathologic factors.

## 4 Discussion

Gastric cancer is one of the most common malignant diseases.^[1–3]^ Although many methods are used to evaluate the characteristics and prognosis of gastric cancer, the TNM staging classification is the standard that is acknowledged by clinical doctors.^[9,10]^ The TNM staging system for gastric cancer classifies gastric cancer according to the depth of tumor invasion (T stage), regional lymph node status (N stage), and the existence of distal metastasis (M stage).^[9,10]^ In the past decades, there have been differences in the classification of gastric cancer between the East and West. However, recent classifications of gastric cancer published by the UICC/AJCC and JGCA achieved consensus in the year 2010. Previously, scholars paid more attention to the relationship between the lymph node or serosa status and patient prognosis.^[6,7]^ More recently, scholars have understood more about the characteristics of gastric cancer. Some studies have focused on subclassification of the T2 stage. In this study, we analyzed the pT2 gastric patients and grouped them into 2 subgroups, the sMP (inner circular muscle) group and dMP (outer longitudinal muscle) group. The dMP group had a higher number of metastatic lymph nodes than the sMP group, 2.1 ± 2.9 vs 3.6 ± 5.7, respectively, *P* = 0.027. In the survival analysis, the depth of tumor invasion, lymph node metastasis, and postoperative chemotherapy were independent, poor prognostic risk factors for the OS outcomes. Specifically, for patients without lymph node metastasis, the sMP group had better survival outcomes than the dMP group (*P* = 0.004).

According to the latest gastric cancer TNM staging system, the T stage is characterized according to the depth of tumor invasion. When reviewing the 7th TNM staging system edition description of gastric cancer, we found that there is no subclassification of the pT2 stage.^[9,10]^ However, the muscularis propria layer consists of 2 different muscle layers. Meanwhile, we found that the pT2 stage includes tumors that invade into the muscularis propria and subserosa layers from the previous gastric cancer TNM classification.^[16]^ We also found that some previous studies analyzed the clinicopathological characteristics and prognosis of T2 stage patients using the previous staging system, which included tumors in the subserosa stage.^[12–15]^ By contrast, this study purely included and analyzed gastric cancer patients with tumors invading the muscularis propria.

For gastric cancer, the tumor size, differentiation degree, lymph node metastasis, extranodal metastasis, and survival outcomes of patients were closely associated with the depth of tumor invasion.^[18–21]^ A previous study reported that the tumor characteristics of gastric cancers invading into the muscularis propria layer are an intermediate between early and advanced cancers.^[22]^ Liu et al^[19]^ analyzed 442 pT2 gastric cancer patients and reported that the depth of tumor invasion, tumor size, and capillary invasion were independent predictive factors for lymph node metastasis. Bu et al found similar results as in a previous study.^[19,23]^ In terms of the tumor characteristics, previous studies have reported that the rate of the lymph node metastasis for T2 stage gastric cancer patients is approximately 40% to 50%, which is significantly higher than for early gastric cancer and lower than for T3/T4 stage gastric cancer patients.^[19,22–24]^ In our study, the rate of lymph node metastasis was 59.7% of T2 stage patients, and this result was similar to that in previous studies. Moreover, Park et al^[15]^ reported that the rates of lymph node metastasis in the sMP group was 53.8% and 71.0% for dMP/SS patients; Sun et al^[12]^ presented that the lymph node metastasis rate was 53.6% in the sMP group, 67.4% in the dMP group, and 75.3% in the SS group. In the present study, the dMP group had a higher rate of lymph node metastasis than the sMP group, 3.6 ± 5.7 vs. 2.1 ± 2.9, respectively, *P* = 0.027. It is interesting that there was no significant difference between the 2 groups in terms of the proportion of pN stages (*P* = 0.228). Additionally, the lymph node metastasis rates for the sMP and dMP groups were 55.1% and 64.4%, respectively, *P* = 0.202. These results were similar as in a previous study. For tumors invading the muscularis propria layer of the stomach, the degree of lymph node metastasis may not be sufficient to significantly change the pN stage between the sMP and dMP groups with the present TNM classification.

However, beyond the tumor characteristics, the survival outcomes of the patients were more powerful evidence for exploring potential differences in the tumor subclassification. Previous studies have successfully demonstrated that pT2a (sMP) gastric cancer patients have significantly better survival outcomes than pT2b (dMP/SS) stage patients.^[14,15]^ However, these studies adopted the previous TNM classification and included subserosa tumors in the pT2b groups, which are now classified as the pT3 stage in the latest TNM classification.^[10,16]^ Park et al^[15]^ observed that the age, pT, and pN stages were independent prognostic factors for gastric cancer patients with pT2 staging. By contrast, Sun et al^[12]^ observed that sMP tumor patients had significantly better survival outcomes than dMP and SS tumor patients, whereas the dMP and SS tumor patients had similar outcomes. In our study, patients in the sMP group had a better prognosis than those in the dMP group according to univariate survival analysis (*P* = 0.020) and multivariate survival analysis (*P* = 0.050). Meanwhile, the pN stage might be another factor beyond the tumor invasion depth that can influence the survival outcomes. A previous study demonstrated that pT2a stage gastric cancer patients had a significantly better prognosis than pT2b stage patients at the pN0, pN1, and pN2 stages, but they had comparable survival outcomes at the pN3 stage.^[14]^ In our study, sMP stage patients had better survival outcomes than dMP patients at the pN0 stages, but not at the pN1–3 stages, which is in agreement with previous studies. Therefore, differences in the survival outcomes between the sMP and dMP tumor groups may not merely depend on the depth of tumor invasion. Lymph node metastasis may another factor influencing the survival. According to the results of our study, for pT2 stage gastric cancers with pN0 stages, the tumor depth of invasion had a bigger effect on the survival than the pN stage, which was not the case for patients with pN1–3 stage tumors.

Postoperative adjuvant chemotherapy is the standard treatment strategy for advanced stage gastric cancer patients who have undergone radical gastrectomy.^[4]^ Postoperative adjuvant chemotherapy improves the prognosis of advanced gastric cancer patients.^[25,26]^ According to the gastric cancer guidelines published by the National Comprehensive Cancer Network (NCCN),^[27]^ postoperative chemotherapy is recommended for pathological T2 stage gastric cancer patients with lymph node metastasis or the N0 stage with high risk factors.^[28]^ Therefore, the proportion of patients treated with postoperative chemotherapy is relatively lower than reported in another study.^[29]^ Univariate and multivariate survival analyses were performed and demonstrated that chemotherapy was an independent prognostic risk factor. In the present study, subgroup analysis of the chemotherapy status demonstrated that there were different results in the OS outcomes of nodal positive patients between the 2 groups. However, these results may be explained by the limitation of the small sample size and that few patients underwent postoperative chemotherapy. These are limitations in the present study that must be clarified.

As a retrospective study, this study has some limitations. First, this is a single center retrospective study with a small sample size. These limitations may reduce the statistical efficiency, and large sample size, multicenter studies are expected to validate the preliminary findings of this study. Second, because of the limitation of retrospective study, we did not analyze the relationship between the tumor biomarkers (Her-2 or Ki-67, etc.) and the depth of tumor invasion. This is another major limitation of this study. In spite of these limitations, this study successfully demonstrated the difference between sMP and dMP gastric cancer patients, which may further help with the TNM stage classification.

## 5 Conclusions

For the muscularis propria gastric cancer patients, there were differences in the clinicopathological characteristics and survival outcomes for sMP and dMP tumors. In spite of these differences, it remains pending whether the pT2 stage should be subclassified into the sMP (pT2a) and dMP (pT2b) stages. Future large sample size, multicenter studies are expected to validate our findings.

## References

[1] Torre LA Bray F Siegel RL Global cancer statistics, 2012. *CA Cancer J Clin* 2015; 65:87–108. 25651787 10.3322/caac.21262

[2] Ferlay J Shin HR Bray F Estimates of worldwide burden of cancer in 2008: GLOBOCAN 2008. International journal of cancer. *J Int Cancer* 2010; 127:2893–2917. 10.1002/ijc.25516 21351269

[3] Colquhoun A Arnold M Ferlay J Global patterns of cardia and non-cardia gastric cancer incidence in 2012. *Gut* 2015; 64:1881–1888. 25748648 10.1136/gutjnl-2014-308915

[4] Shen L Shan YS Hu HM Management of gastric cancer in Asia: resource-stratified guidelines. *Lancet Oncol* 2013; 14:e535–e547. 24176572 10.1016/S1470-2045(13)70436-4

[5] Sasako M Gastric cancer eastern experience. *Surg Oncol Clin N Am* 2012; 21:71–77. 22098832 10.1016/j.soc.2011.09.013

[6] Nashimoto A Akazawa K Isobe Y Gastric cancer treated in 2002 in Japan: 2009 Annual Report of the JGCA Nationwide Registry. *Gastric Cancer* 2013; 16:1–27. 22729699 10.1007/s10120-012-0163-4 PMC3549249

[7] Zhang WH Chen XZ Liu K Outcomes of surgical treatment for gastric cancer patients: 11-year experience of a Chinese high-volume hospital. *Med Oncol* 2014; 31:150 doi: 10.1007/s12032-014-0150-1. 25112468 10.1007/s12032-014-0150-1

[8] Hohenberger P Gretschel S Gastric cancer. *Lancet* 2003; 362:305–315. 12892963 10.1016/s0140-6736(03)13975-x

[9] Washington K 7th edition of the AJCC cancer staging manual: stomach. *Ann Surg Oncol* 2010; 17:3077–3079. 20882416 10.1245/s10434-010-1362-z

[10] Japanese Gastric Cancer Association.. Japanese classification of gastric carcinoma: 3rd English edition. *Gastric Cancer* 2011; 14:101–112. 21573743 10.1007/s10120-011-0041-5

[11] Huang CM Wang HM Zheng CH Tumor size as a prognostic factor in patients with node-negative gastric cancer invading the muscularis propria and subserosa (pT2-3N0M0 stage). *Hepatogastroenterology* 2013; 60:699–703. 23159390 10.5754/hge12733

[12] Sun Z Zhu GL Lu C A novel subclassification of pT2 gastric cancers according to the depth of muscularis propria invasion: superficial muscularis propria versus deep muscularis propria/subserosa. *Anna Surg* 2009; 249:768–775. 10.1097/SLA.0b013e3181a3df77 19387327

[13] Bilici A Dane F Seker M Is subdivision of pT2 tumors superior to lymph node metastasis for predicting survival of patients with gastric cancer? Review of 224 patients from four centers. *Digest Dis Sci* 2011; 56:3226–3234. 21660487 10.1007/s10620-011-1721-z

[14] Lu Y Liu C Zhang R Prognostic significance of subclassification of pT2 gastric cancer: a retrospective study of 847 patients. *Surg Oncol* 2008; 17:317–322. 18586486 10.1016/j.suronc.2008.05.005

[15] Park DJ Kong SH Lee HJ Subclassification of pT2 gastric adenocarcinoma according to depth of invasion (pT2a vs pT2b) and lymph node status (pN). *Surgery* 2007; 141:757–763. 17560252 10.1016/j.surg.2007.01.023

[16] Japanese Gastric Cancer Association. Japanese classification of gastric carcinoma—2nd English edition—response assessment of chemotherapy and radiotherapy for gastric carcinoma: clinical criteria. *Gastric Cancer* 2001; 4:1–8. 11706621 10.1007/s101200100009

[17] Japanese Gastric Cancer Association. Japanese gastric cancer treatment guidelines 2010 (ver. 3). *Gastric Cancer* 2011; 14:113–123. 21573742 10.1007/s10120-011-0042-4

[18] Nitti D Marchet A Mocellin S Prognostic value of subclassification of T2 tumours in patients with gastric cancer. *Br J Surg* 2009; 96:398–404. 19283740 10.1002/bjs.6487

[19] Liu X Long Z Cai H Analysis of lymph node metastasis correlation with prognosis in patients with T2 gastric cancer. *PLoS ONE* 2014; 9:e105112. 25136920 10.1371/journal.pone.0105112 PMC4138144

[20] Chen S Cai MY Chen YB Serosa-penetration in human T4aN0M0 gastric carcinoma correlates with worse prognosis after D2 gastrectomy. *Chin Med J* 2012; 125:1158–1162. 22613547

[21] Marutsuka T Shimada S Shiomori K Mechanisms of peritoneal metastasis after operation for non-serosa-invasive gastric carcinoma: an ultrarapid detection system for intraperitoneal free cancer cells and a prophylactic strategy for peritoneal metastasis. *Clin Cancer Res* 2003; 9:678–685. 12576435

[22] Otsuji E Kuriu Y Ichikawa D Characteristics of gastric carcinoma invading the muscularis propria. *J Surg Oncol* 2005; 92:104–108. 16231368 10.1002/jso.20345

[23] Bu Z Zheng Z Li Z Lymphatic vascular invasion is an independent correlated factor for lymph node metastasis and the prognosis of resectable T2 gastric cancer patients. *Tumour Biol* 2013; 34:1005–1012. 23292920 10.1007/s13277-012-0637-3

[24] Yokota T Kunii Y Teshima S Gastric cancer with invasion limited to the muscularis propria. *Int Surg* 1999; 84:7–12. 10421010

[25] Noh SH Park SR Yang HK Adjuvant capecitabine plus oxaliplatin for gastric cancer after D2 gastrectomy (CLASSIC): 5-year follow-up of an open-label, randomised phase 3 trial. *Lancet Oncol* 2014; 15:1389–1396. 25439693 10.1016/S1470-2045(14)70473-5

[26] Sasako M Sakuramoto S Katai H Five-year outcomes of a randomized phase III trial comparing adjuvant chemotherapy with S-1 versus surgery alone in stage II or III gastric cancer. *J Clin Oncol* 2011; 29:4387–4393. 22010012 10.1200/JCO.2011.36.5908

[27] https://www.nccn.org/professionals/physician_gls/pdf/gastric.pdf.

[28] Du C Zhou Y Huang K Defining a high-risk subgroup of pathological T2N0 gastric cancer by prognostic risk stratification for adjuvant therapy. *J Gastrointest Surg* 2011; 15:2153–2158. 21938559 10.1007/s11605-011-1684-6

[29] Messager M Lefevre JH Pichot-Delahaye V The impact of perioperative chemotherapy on survival in patients with gastric signet ring cell adenocarcinoma: a multicenter comparative study. *Ann Surg* 2011; 254:684–693.discussion 693. 22005144 10.1097/SLA.0b013e3182352647

